# The epidemiology of heart failure in the general Australian community - study of heart failure in the Australian primary carE setting (SHAPE): methods

**DOI:** 10.1186/s12889-020-08781-8

**Published:** 2020-05-11

**Authors:** Richard Whaddon Parsons, Danny Liew, A. Munro Neville, Ralph G. Audehm, Deepak Haikerwal, Peter Piazza, Kevin Lim, Andrew P. Sindone

**Affiliations:** 1 AusTrials, Brisbane, Australia; 2 School of Public Health and Preventive Medicine, Monash University, Melbourne, Australia; 3 Dept of General Practice, University of Melbourne, Melbourne, Australia; 4 Heartwest, Melbourne, Australia; 5 Five Dock Family Medical Practice, Sydney, Australia; 6 Novartis Pharmaceuticals Pty. Ltd, Sydney, Australia; 7 Director Heart Failure Unit and Department of Cardiac Rehabilitation, Concord Hospital, Sydney, Australia

**Keywords:** Retrospective, Heart failure, HF, Primary care, Australia, Epidemiology, Prevalence, Incidence, Demographics, SHAPE

## Abstract

**Background:**

There is a paucity of information on the epidemiology of heart failure (HF) in Australia. The Study of Heart failure in the Australian Primary carE setting (SHAPE) study aims to estimate the prevalence and annual incidence of HF in the general Australian community and to describe the demographic and key clinical profile of Australians with HF.

**Methods:**

We undertook a retrospective cohort study based on analysis of non-identifiable medical records of adult patients cared for at 43 general practices between 1 July 2013 and 30 June 2018. Data were extracted from coded (diagnosis, pathology and prescription fields) and uncoded fields (clinical notes) in the medical records. The latter searches of free text looked for common synonyms relevant to HF. The population was stratified into three groups based on a hierarchy of selection criteria: (1) definite HF, (2) probable HF and (3) possible HF. The prevalence and annual incidence of HF were calculated, along with 95% confidence intervals.

**Results:**

The practices provided care to 2.3 million individual patients over the five-year study period, of whom 1.93 million were adults and 1.12 million were regular patients. Of these patients 15,468 were classified as having ‘definite HF’, 4751 as having ‘probable HF’ and 33,556 as having ‘possible HF’. A further 39,247 were identified as having an aetiological condition associated with HF.

A formal HF diagnosis, HF terms recorded as text in the notes and HF-specific medication were the most common methods to identify ‘definite’ HF patients. Typical signs and symptoms in combination with a diuretic prescription was the most common method to identify ‘probable HF’ patients. The majority of ‘possible’ HF patients were identified by the presence of 2 or more of the typical signs or symptoms. Dyspnoea was the commonest recorded symptom and an elevated jugular venous pressure the commonest recorded sign.

**Conclusions:**

This novel approach to undertaking retrospective research of primary care data successfully analysed a combination of coded and uncoded data from the electronic medical records of patients routinely managed in the GP setting. SHAPE is the first real-world study of the epidemiology of HF in the general Australian community setting.

## Background

Currently, heart failure (HF) is estimated to affect 480,000 Australians, with over 60,000 new diagnoses made every year [1]. The continued ageing of the Australian population is expected to further increase the burden of HF on the healthcare system in general and on primary care more specifically [2]. Of patients hospitalised with HF, only 20% are subsequently enrolled in hospital-based disease management programs, with access especially limited in regional and remote communities [3]. The remaining 80% of patients are mostly reliant upon their general practitioners (GPs) for ongoing management of their HF.

In Australia, GPs are the healthcare providers who co-ordinate and monitor the care of patients with HF, including for follow up and referral to specialist care, such as cardiologists and renal physicians [4]. To date, little information has been gathered regarding the prevalence and incidence of HF in the general Australian community, as well as the demographic and clinical profiles of patients with HF. Such information is important for healthcare planning, as well as for establishing a baseline against which to compare future epidemiological data. Furthermore, insight is needed into areas in which the management of HF can be improved. There are data suggesting that many patients with HF are not optimally treated in primary care, with the majority of patients not being on the most appropriate therapies or at target doses [5, 6].

The Study of Heart failure in the Australian Primary carE setting (SHAPE) is a retrospective cohort study of primary care data that seeks to estimate the prevalence and annual incidence of HF in the Australian general community and to describe the demographic and key clinical profile of Australians with prevalent HF. The present article describes the methods undertaken in SHAPE and provides an overview of the main epidemiological findings.

## Methods

We undertook a retrospective cohort study based on analysis of existing medical records of adult patients cared for at participating general practices between 1 July 2013 and 30 June 2018. All practices provided fully subsidised care to their patients (which is known as ‘bulk-billing’). Participating practices were those within the Healius network (previously known as Primary Health Care) that used Medical Director software - this group comprised 43 centres from a network of 71. The remaining 28 practices were using software other than Medical Director and so data were not available for extraction and analysis. All centres in the network are transitioning over to the Medical Director software according to a schedule. There were no other regional or socioeconomic differences between those included in our study and those omitted for software reasons.

The use of prescribing and electronic health records in Australian general practices has been widely adopted such that by 2005, Australian general practice had achieved near-universal clinical computerisation [7, 8]. Medical Director software is one of two dominant providers of practice software in Australia, providing 4300 GP practices and 13,600 GP users with its practice software [9].

To identify patients with HF, a search of records was undertaken using Structured Query Language (SQL). The list of screening words was broad so that cases would be unlikely be missed. Search terms to identify a cohort for extraction and full analysis included HF diagnostic terms, HF-specific medication use, signs and symptoms of HF, pathology test results indicative of HF, the diagnosis of an aetiological condition for HF (Table 1) and a referral for cardiac imaging, principally echocardiography. These criteria were developed with expert opinion advice or from current Australian HF evidence-based guidelines [10]. All patients visiting the practices (with and without heart failure) were included and heart failure hospitalisation was not a prerequisite for being included in the study.

**Table 1:** Comorbidities that are an aetiological condition for HF

Coronary artery disease	Lysosomal storage disease
Coronary heart disease	Fabry disease
Peripheral vascular disease	Thyroid insufficiency
Peripheral arterial disease	Hyperactive thyroid
Myocardial infarction	Thyrotoxicosis
Unstable angina	Hashimoto’s thyroiditis
Acute coronary syndrome	Grave’s disease
Cardiac dysrhythmia / cardiac arrhythmia	Cushing’s syndrome
Atrial fibrillation and atrial flutter	Adrenal insufficiency
Ischaemic heart disease	Diabetes mellitus
Myocardial ischaemia	Pheochromocytoma
Microvascular disease	Malnutrition
Myocyte stunning	Hypertension
Myocyte hibernation	Pericarditis
Myocarditis	Pericardial constriction
Cardiomyopathy	Pericardial effusion
Heart valve disease	Hypereosinophilic syndromes
Alcoholism	Endomyocardial fibrosis
Cardiac malignancy	Fibroelastosis
Arteriovenous fistula	Anaemia
Amyloidosis	Sepsis
Sarcoidosis	Paget disease
Haemochromotosis	Renal failure
Iron overload	Iatrogenic fluid overload
Glycogen storage disease	Thiamine deficiency
	Vitamin B deficiency

Source: SHAPE Project Collaborators Expert Opinion, September 2018

The cases were de-identified, removing all potentially identifiable data from the records, then provided to the researchers for analysis. Data were extracted from the following fields in the medical records: diagnosis, reason for presentation, prescriptions, vital signs, pathology results, specialist referrals and clinical notes. Chronic disease management item numbers billed to Medicare were also extracted. Each patient was allocated a unique study number so that re-identification would be possible by Healius for future scrutiny of records for any reason (for example, missing data). This allowed records belonging to the same patient to be linked through time so that GP visits and management for each patient could be identified.

To ensure data integrity, consistency and completeness of the data extraction, a detailed quality control process was performed. A registered nurse who was an experienced study coordinator employed by Healius examined the records of a random sample of 50 identified patients to ensure that the query collected the correct data from the correct patients. The study coordinator also performed a disease register search of HF to make sure that the query did not omit from the extract any potential HF patients. This quality control process confirmed that the data extraction produced the correct patient level results and showed that the query was comprehensive so that HF patients were very unlikely to be omitted.

The study’s primary endpoints were the prevalence and incidence of HF, stratified by age and gender, and standardised to the 2017 Australian population. We also sought to determine the demographics of the HF population and their clinical characteristics, including aetiological factors, comorbidities, symptoms of HF, examination findings and medication use. Other factors examined included the proportion receiving HF medications, the proportion receiving medications that are contraindicated in HF, the frequency of GP visits, the use of GP chronic disease management Medicare services, the use of mental health services, and the frequency of referrals from GPs to specific types of specialists. In the primary analyses, data comprised only ‘active’ patients; that is those patients who visited the medical centres at least three times over a two-year period [11].

Included patients were those who were aged 18 years and above, and who had one or more of the following criteria recorded in their medical record: i) a specific diagnosis of HF (Table 2); ii) were receiving ongoing treatment with a HF-specific medication (Table 3); iii) presented with signs or symptoms of HF (Table 4 and Table 5); or iv) had pathology test results indicative of HF (Table 6 and Table 7). In Australia, the HF-specific medications listed in Table 3 have a ‘Restricted Benefit’ in the Pharmaceutical Benefits Scheme (Australia’s list of subsidised medications) to ‘moderate to severe heart failure’. Furthermore, the restriction stipulates that patients must be stabilised on conventional therapy, which must include an angiotensin converting enzyme inhibitor or angiotensin II antagonist, if tolerated [12]. In the search of text fields, certain criteria were selected for common synonyms, which are listed in Additional file 1 Appendix - Free text search terms. If certain words preceded the selected words in the notes, then the condition was considered not to be present in those notes. For example, if there was a mention of ‘shortness of breath (SOB)’, but this was preceded by ‘No’, ‘Nil’, or ‘denies’, then SOB was considered not to be a problem for the patient at that time.

**Table 2:** Heart failure diagnosis terms

Past History Diagnosis or Reason for Presentation Keywords for Inclusion	
Heart failure	Systolic heart failure
Chronic cardiac failure	Systolic dysfunction
Chronic heart failure	Diastolic heart failure
Congestive cardiac failure	Diastolic dysfunction
CCF	
Congestive heart failure	HFrEF / Heart failure with reduced ejection fraction
CHF	
Cardiac failure	HFpEF / Heart failure with preserved ejection fraction
Chronic heart failure	Pulmonary oedema
Left ventricular failure	Ischaemic cardiomyopathy
Right ventricular failure	Dilated cardiomyopathy (but excluding hypertrophic cardiomyopathy)

Source: SHAPE Project Collaborators Expert Opinion, September 2018

**Table 3:** Heart failure specific medications

Ivabradine	
Ethacrynic acid	
Eplerenone	
Bisoprolol	
Nebivolol	
Carvedilol	
Metoprolol succinate (HF doses only^a^)	
Sacubitril / Valsartan	

Source: SHAPE Project Collaborators Expert Opinion, September 2018

In Australia, these medications have a restricted use benefit in the Pharmaceutical Benefits Scheme to ‘moderate to severe heart failure’ only. For example: https://www.pbs.gov.au/medicine/item/8733P for metoprolol succinate

^a^Doses 23.75 mg, 47.5 mg, 95 mg, 190 mg (controlled release)

**Table 4:** Typical and specific symptoms and signs of heart failure

Symptoms of heart failure	Signs of heart failure
Dyspnoea (usually with exertion)	Elevated jugular venous pressure
Orthopnoea	Hepatojugular reflux
Paroxysmal nocturnal dyspnoea	Third heart sound
	Laterally displaced apex beat

Source: Atherton J, Sindone A, De Pasquale C, et al. National Heart Foundation of Australia and Cardiac Society of Australia and New Zealand Guidelines for the prevention, detection and management of heart failure in Australia 2018

**Table 5:** Less typical symptoms and signs of heart failure

Symptoms	Signs
Nocturnal cough	Weight gain (> 2 kg/week)
Bendopnoea	Peripheral oedema (ankle, sacrum)
	Pulmonary crackles

Source: Atherton J, Sindone A, De Pasquale C, et al. National Heart Foundation of Australia and Cardiac Society of Australia and New Zealand Guidelines for the prevention, detection and management of heart failure in Australia 2018

**Table 6:** Pathology tests and indicative cut-offs for definite heart failure

	Test name	
	BNP	NT-ProBNP
Heart failure rule-out	< 100 pg/mL	< 300 pg/mL
Heart failure rule-in	> 400 pg/mL	> 450 pg/mL age < 50 yrs
		> 900 pg/mL age 50–75 yrs
		> 1800 pg/mL age > 75 yrs

Source: Atherton J, Sindone A, De Pasquale C, et al. National Heart Foundation of Australia and Cardiac Society of Australia and New Zealand Guidelines for the prevention, detection and management of heart failure in Australia 2018

**Table 7:** Pathology cut-offs suggestive but inconclusive of heart failure (probable HF)

	Test name	
	BNP	NT-ProBNP
Heart failure rule-out	< 100 pg/mL	< 300 pg/mL
Heart failure rule-in(probable HF)	> = 100 pg/mL	300–450 pg/mL age < 50 yrs
		300–900 pg/mL age 50–75 yrs
		300–1800 pg/mL age > 75 yrs

Source: SHAPE Project Collaborators Expert Opinion, September 2018

The search term ‘PND’ was found to produce a lot of false positive results (also being used for other conditions, such as post-natal depression, and post-nasal drip). A review of 2000 records with ‘PND’ was undertaken and this included 1151 with nasal symptoms, 659 with upper respiratory tract infection (URTI), 515 with sinusitis, 169 with lower respiratory tract infection (LRTI) and 63 with depression. However, the term ‘PND’ was still included, but non-HF causes were excluded and further supporting evidence (ejection fraction data, BNP data, or loop diuretic use) was required in order for a case to be classified as definite or probable HF.

The analysis assessed the number and combinations of relevant terms and cut-off criteria in a hierarchical approach. The population was then stratified into three groups based on a hierarchy of selection criteria: (1) definite HF, (2) probable HF and (3) possible HF (Table 8). The eligibility criteria for ‘definite HF’ were: HF coded in the field of diagnosis codes; any mention of HF diagnoses in the free text fields; prescription of HF-specific drugs; BNP/ NT-ProBNP above HF cut-offs; recorded ejection fraction (EF) < 40%, EF ≥40 - < 50% and typical symptoms and signs recorded in the notes; and EF ≥40 - < 50% & use of a loop diuretic The criteria for ‘probable HF’ were recorded EF ≥40 - < 50%, or typical symptoms and signs of HF recorded in the notes & any of the following: BNP/ NT-ProBNP in the inconclusive ranges, use of a loop diuretic, or documented EF > 50%.

**Table 8:** Criteria for stratification and number of patients by group

		Number of patients	
Group	Criteria	All	Active only
1 Patients who definitively had HF:	• HF diagnosis recorded in the diagnosis/condition section, or	3193	3026
	• HF diagnosis recorded or as free text in the notes, or	8744	8103
	• Having had an HF-specific medication, or	4773	4132
	• EF reduced (from free text in the notes), or	144	137
	• BNP/ NT-ProBNP above HF cut-offs, or	50	45
	• Recorded ejection fraction (EF) < 40%, or	12	11
	• EF ≥40 - < 50% and typical symptoms and signs recorded in the notes, or	10	10
	• EF ≥40 - < 50% & use of a loop diuretic	4	4
2 Patients who had a probable diagnosis of HF:	• EF ≥40 - < 50%, or	19	19
	• Typical symptoms and signs recorded in the notes AND any of the following:		
	• BNP/ NT-ProBNP in the inconclusive ranges	38	38
	• Use of a loop diuretic	4754	4635
	• Documented EF > 50%	62	60
3 Patients where HF was possible:	• Two or more of the less typical symptoms and signs recorded either in the diagnosis/condition section or in the notes, or	109	107
	• Typical symptoms and signs recorded either in the diagnosis/condition section or in the notes (only), or	36,224	33,278
	• EF > 50% or EF found in notes, but no percentage recorded, or	100	100
	• BNP/ NT-ProBNP in the inconclusive ranges	84	71

In Australia, the Federal Government mandates through the Pharmaceutical Benefits Scheme (PBS) that the HF-specific drugs used in our search and analysis are for to be prescribed for the management of heart failure only. General practitioners are unlikely to stray from these restrictions – education must be in line with the PBS listing and Medicare can perform audits on GPs’ practices. Also, as HF is a clinical diagnosis that can also be inferred from response to treatment, it would be highly likely that patients with the constellation of symptoms described plus prescriptions for diuretic medication/s would have heart failure, even if no specific diagnosis has been entered or other more specific HF medications not initiated.

Data analysis was conducted using SAS for windows (version 9.4). For laboratory and other data, the most recent measurement for each patient of each parameter was selected for analysis. If any of the selected drugs were taken at any time by a patient during the whole period under study, then that patient was identified as having been prescribed that drug. Medications prescribed following the diagnosis of HF was also reviewed. Referrals (to a cardiologist, endocrinology or renal physician) were recorded for a patient only if the referral occurred around the time of diagnosis of HF, or later. That period started one month prior to HF diagnosis and then onwards. This presumed that the referral to the specialist was the time when the GP was suspecting HF and was seeking specialist involvement. We also assessed referrals starting from seven months prior to diagnosis, which allowed for patients to have been seen by a specialist, provided with six months of prescriptions and so only needed a GP consultation after this period. In this case, the diagnosis may only appear in the GP records up to a maximum of seven months after the specialist visit.

The point prevalence and annual incidence of HF were calculated, along with their 95% confidence intervals. From the age- and gender-specific rates of HF, and estimates of the Australian population in these subgroups, prevalence and incidence were age-standardised to the 2017 Australian population overall, and by gender.

The calculation of prevalence and incidence of HF involved only ‘active’ patients; that is, those patients with at least three visits per two-year period [11]. This approach avoided the under-estimation of prevalence and incidence that would have otherwise arisen from over-inflation of the denominator data by one-off or infrequent GP visits. Such visits would be more common in bulk billing centres. Furthermore, among people who are not regular patients of the centres, medical records may not contain sufficient information on which to assess the presence of a HF diagnosis. In secondary analyses, denominators were estimated from the total number of patients seen at the participating GP clinics during each calendar year for the period under study. Overall prevalence within gender and age groups was calculated, along with the proportion of cases within each of the gender and age-groups.

The numerator for the prevalence of HF was obtained by tabulating the numbers of HF cases by age group and gender over the five-year period under study. Annual incidence of HF was reported similarly to prevalence, except that only new cases were included, based on the date of first diagnosis of HF. In an attempt to remove from the file the cases with pre-existing HF, we identified cases where the diagnosis of definite and probable HF was made or was present during the first year of data collection and removed these from the file. This meant that cases which remained in the incidence calculation had no mention of HF during the first year of the data collection.

## Results

**Fig. 1:**
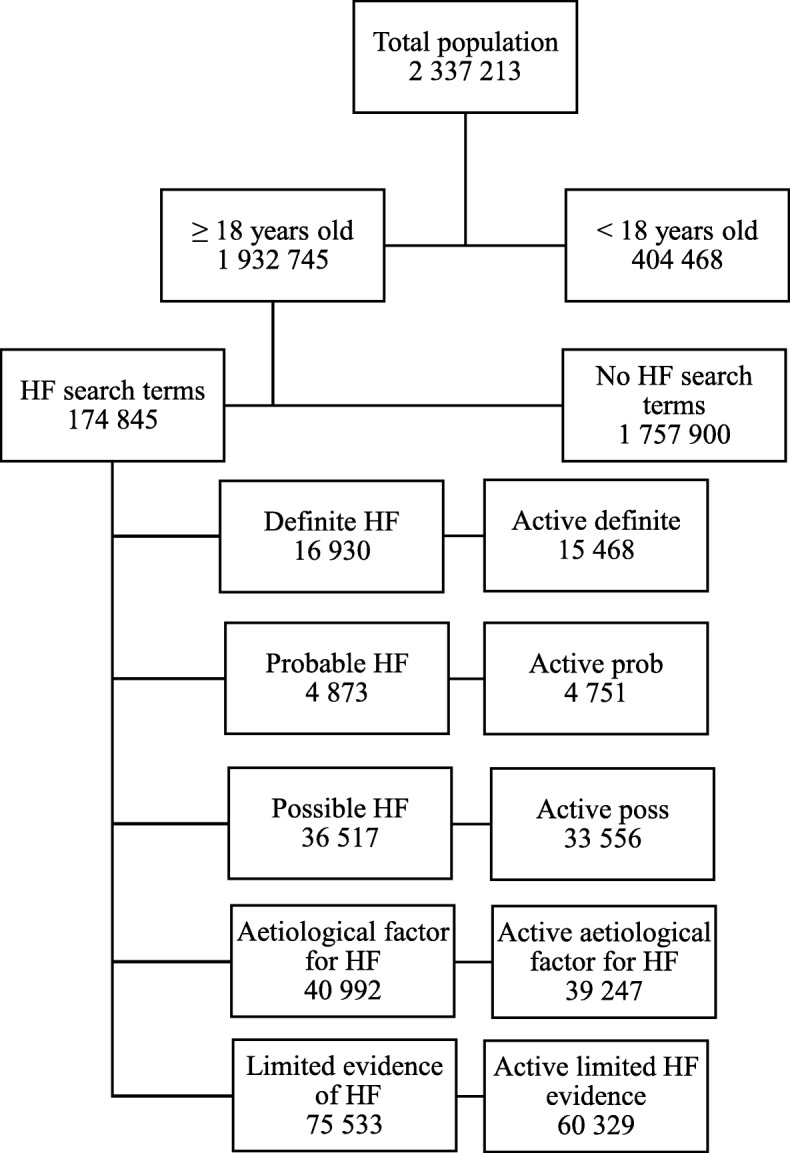
Flow of patients and allocation to groups

The practices provided care to 2.3 million individual patients over the five-year period, of which 1.93 million were aged 18 years and above. Among these, 1.12 million were ‘active’ patients [11]. Based on the presence of one or more of the HF search terms (Table 2 to Table 7), full clinical data on 174,845 patients were extracted for further analyses. Of these patients, and based on the hierarchy of selection criteria (Table 8), 16,930 were classified as having ‘definite HF’, 4873 as having ‘probable HF’ and 36,517 as having ‘possible HF’ (Fig. 1). A further 40,992 were identified as having an aetiological condition associated with HF. The remaining 75,533 were initially identified for analysis on the basis of signs or symptoms of HF recorded as free text in the notes only. As these patients had limited evidence of HF, they were excluded from further analyses. The most frequent signs or symptoms from this group are displayed in Table 9, with paroxysmal nocturnal dyspnoea (PND), ankle oedema and weight gain being the most common.

**Table 9:** Frequency of symptoms and signs of heart failure recorded in patients who had limited evidence of HF and were excluded from analysis

Symptoms or sign	Frequency recorded
PND	26.1%
Ankle oedema	23.1%
Weight gain	19.7%
Rales	8.7%
Ankle swelling	6.0%
Crepitations	4.4%
Leg swelling	4.0%
Pitting oedema	1.7%
Leg oedema	1.0%
Peripheral oedema	1.0%

For the active patients, 15,468 were classified as having ‘definite HF’, 4751 as having ‘probable HF’ and 33,556 as having ‘possible HF’ (Fig. 1). A further 39,247 were identified as having an aetiological condition associated with HF.

It was not possible to identify the amount nor rate of weight gain to satisfy the ‘> 2 kg/week’ criterion and some other signs and symptoms, such as peripheral oedema and crepitations, were only included in the classification of possible HF if more than one of these signs or symptoms had been recorded.

The flow of patients in the search and evaluation process is shown in Fig. 1.

In the primary analysis of active patients, a formal HF diagnosis (3026, 19.5%), HF terms recorded as free text in the notes (8103, 52.4%) and HF-specific medication prescriptions (4132, 26.7%) were the most common methods to identify ‘definite’ HF patients (Table 8). Typical signs and symptoms of HF in combination with a diuretic prescription (4635, 97.6%) was the most common method to identify ‘probable HF’ patients. The vast majority of ‘possible’ HF patients (33,278, 99.2%) were identified by the presence of 2 or more of the typical signs or symptoms of HF (Table 8).

The most commonly recorded diagnostic terms for HF in the active population were ‘congestive heart failure’ (4393, 21.7%), ‘heart failure’ (2177, 10.8%) and ‘cardiac failure’ (674, 4.7%). Other frequently used terms included ‘diastolic dysfunction’, ‘pulmonary oedema’ and ‘cardiomyopathy’ (Table 10).

**Table 10:** Top 10 diagnosis terms recorded in the definite HF population

	Diagnostic terms recorded in the notes	Number	Percent
All patients			
1	Congestive heart failure	4663	27.5%
2	Heart failure	2457	14.5%
3	Cardiac failure	723	4.3%
4	Heart failure & Congestive heart failure	672	4.0%
5	Diastolic dysfunction	370	2.2%
6	Pulmonary oedema	364	2.2%
7	Cardiomyopathy	217	1.3%
8	Cardiac failure & Congestive heart failure	214	1.3%
9	Ventricular failure	194	1.1%
10	Heart failure & Cardiac failure	169	1.0%
Active patients			
1	Congestive heart failure	4393	28.4%
2	Heart failure	2177	14.1%
3	Cardiac failure	674	4.4%
4	Heart failure & Congestive heart failure	639	4.1%
5	Diastolic dysfunction	356	2.3%
6	Pulmonary oedema	331	2.1%
7	Cardiomyopathy	208	1.3%
8	Cardiac failure & Congestive heart failure	208	1.3%
9	Ventricular failure	181	1.2%
10	Heart failure & Cardiac failure	160	1.0%

Terms such as ‘HFrEF’ and ‘HFpEF’, which have been in use for a few years, were not commonly noted. We found 19 records of these terms (one record of HFrEF, and 18 records of HFpEF).

The most commonly prescribed HF-specific medications to the active ‘definite’ and ‘probable’ HF population were the beta blockers bisoprolol (3783, 18.7%), carvedilol (957, 4.7%) and nebivolol (736, 3.6%), Table 11.

**Table 11:** Top 10 HF-specific medications prescribed in the definite HF population

	HF-specific medications recorded in the notes	Number	Percent
All patients			
1	Bisoprolol	4175	24.7%
2	Carvedilol	1093	6.5%
3	Nebivolol	793	4.7%
4	Metoprolol succinate	646	3.8%
5	Ivabradine	171	1.0%
6	Bisoprolol & Sacubitril	113	0.7%
7	Bisoprolol & Carvedilol	110	0.6%
8	Eplerenone & Bisoprolol	110	0.6%
9	Eplerenone	77	0.5%
10	Bisoprolol & Nebivolol	59	0.3%
Active patients			
1	Bisoprolol	3783	24.5%
2	Carvedilol	957	6.2%
3	Nebivolol	736	4.8%
4	Metoprolol succinate	554	3.6%
5	Ivabradine	153	1.0%
6	Bisoprolol & Sacubitril	111	0.7%
7	Bisoprolol & Carvedilol	107	0.7%
8	Eplerenone & Bisoprolol	101	0.7%
9	Eplerenone	72	0.5%
10	Bisoprolol & Nebivolol	56	0.4%

Signs and symptoms of HF were extracted from the free text of the consultation notes. Dyspnoea was by far the commonest recorded symptom in the active population (9401, 46.5%), followed by the combination of dyspnoea & paroxysmal nocturnal dyspnoea (638, 3.2%), paroxysmal nocturnal dyspnoea (550, 2.7%) and the combination of dyspnoea & orthopnoea (535, 2.6%), Table 12. The commonest recorded sign was elevated jugular venous pressure (JVP) in combination with dyspnoea (117, 0.6%), followed by a displaced apex beat (100, 0.5%) and elevated JVP alone (52, 0.3%), Table 12.

**Table 12:** Top 10 signs and symptoms of HF recorded in the definite and probable HF population

	Sign/symptom combinations recorded in the notes	Number	Percent
All patients			
1	Dyspnoea	9699	44.5%
2	Dyspnoea & PND	646	3.0%
3	PND	561	2.6%
4	Dyspnoea & Orthopnoea	549	2.5%
5	Dyspnoea & displaced apex beat	231	1.1%
6	Orthopnoea	149	0.7%
7	Dyspnoea, Orthopnoea & PND	130	0.6%
8	Dyspnoea & elevated JVP	123	0.6%
9	Displaced apex beat	104	0.5%
10	Elevated JVP	58	0.3%
Active patients			
1	Dyspnoea	9401	46.5%
2	Dyspnoea & PND	638	3.2%
3	PND	550	2.7%
4	Dyspnoea & Orthopnoea	535	2.6%
5	Dyspnoea & displaced apex beat	225	1.1%
6	Orthopnoea	138	0.7%
7	Dyspnoea, Orthopnoea & PND	124	0.6%
8	Dyspnoea & elevated JVP	117	0.6%
9	Displaced apex beat	100	0.5%
10	Elevated JVP	52	0.3%

Among the active population, the crude prevalence of definite or probable HF was 1.815% (95% CIs 1.79–1.84%); and the age-standardised prevalence was 2.199% (95% CIs: 2.168–2.23%). The crude incidence of definite or probable HF was 0.291% per year (95% CIs 0.286–0.296%), and the age-standardised incidence was 0.348% per year (95% CIs: 0.342–0.354%). The estimates of prevalence and incidence suggest that almost 420,000 people were living with HF in Australia in 2017, and over 66,000 new cases of HF occurred that year.

## Discussion

Although medical record systems in the primary care setting can be well-structured, provider compliance with populating the records in accordance with the systems intended structure is variable and often incomplete [13]. We found that over 80% of patients identified as definite HF did not have a HF diagnosis recorded in the diagnosis section of their medical records, although over half (51.6%) of the remainder had a diagnostic term recorded as free text in the consultation notes. Addressing this underuse of diagnostic codes is one of the goals of the Federal Health Department’s new Practice Incentives Program (PIP) Quality Improvement (QI) incentive [14]. In this program, which commenced in August 2019, practices are rewarded for participating in continuous quality improvement activities in partnership with their local Primary Health Network (PHN). The areas chosen for improvement are to be informed by GPs’ clinical information system data, from data collected by the PHN against specified Improvement Measures [14].

Despite a paucity of information on HF, previous studies of the HF epidemiology in Australia have shown consistent findings. In a recent article, investigators applied international data to Australian Bureau of Statistics population figures to estimate the prevalence (2.1%) and annual incidence (0.27%) of HF [1]. These figures were obtained by extrapolating from numbers of hospitalised cases, so that it may not be surprising that the figures presented in this paper were marginally higher than our own estimates. In the USA, prevalence of HF in the United States was reported to be 2.42% in 2012 [15]. A limited number of studies from Asian countries report prevalence estimates in the range 1.26–6.7% [16]. Again, results in these studies were derived from demographic extrapolations of international data, often of hospitalised or post-hospitalised patients. In contrast, our estimates have been determined from medical records obtained from a general practice database that do not rely on hospitalisation as a marker for diagnosis. Accordingly, the patients in our dataset appear to be less co-morbid and on fewer evidence-based medications [17].

### Limitations

As many of the clinics had transitioned from another practice software to Medical Director software over the course of the study period, it is possible that some diagnostic terms were lost in the transition process and not entered into the correct field in the Medical Director software.

Some data in the records are not available for electronic assessment as they are contained in scanned attachments in the systems (e.g. discharge summaries, echocardiogram reports) which may have reduced our ability to identify the presence and severity of heart failure and outcomes (eg rehospitalisation, death). As the point of diagnosis, treatment initiation and performance of key investigations may occur in the hospital setting, some patients may have been reclassified if the full hospital data had been available.

The use of programming methods to search free text for specific keywords is an inexact science. However, a number of records were reviewed manually to refine the search criteria and confirm that commonly appearing misspellings of words were correctly identified. It was not feasible to review a large number of patient notes (there were over 8 million records in total), but we believe that misclassification errors would have occurred infrequently so that the final results should be a good representation of the epidemiology in the Australian community setting.

It is known that approximately 13% of the population do not see their GP annually [18], and the likelihood is that very few of this group would have HF. Therefore, our figures for prevalence and incidence for the whole population may be overestimated, as they are based only on those patients who visit their GP actively.

Finally, in estimating HF incidence, prevalent cases were removed and those that remained for analysis had no mention of HF during the first year. This assumed that participating practices did not inherit new patients with existing HF during the subsequent years.

## Conclusions

This novel approach to undertaking retrospective research of primary care data successfully analysed a combination of coded and uncoded data from the electronic medical records of patients routinely managed in the GP setting.

This has allowed us to produce the first definitive study of the epidemiology of HF in the general Australian community, quantifying the epidemiological characteristics of this population and providing valuable insight into the landscape of HF in Australian primary care, the SHAPE study.

Further analysis will inform on the current care of people with HF and provide guidance of how to improve their management.

A major issue facing such projects in the future is the issue of coding diseases. Our study found that the majority of patients with HF were not clinically coded for HF. Attention needs to be focused on supporting primary care to improve the entry of data into electronic medical records to enable better use and interpretation of these data.

## Supplementary information

**Additional file 1.** Appendix - Free text search terms.

## Data Availability

Data, which are derived from de-identified electronic medical records, are not publicly available and will not be made available to the general public. The data were provided by the participating medical centres belonging to an Australian health care company (Healius Ltd) which de-identified the data, removing all potentially identifiable data from the records, then provided to the researchers for analysis. Access to these data was granted by Healius following independent ethics approval of the study and institutional governance approval.

## Abbreviations

ATC: Anatomical therapeutic chemical
BNP: Brain natriuretic peptide
DoH: Department of Health
EC: Ethic Committee
EF: Ejection fraction
EMR: Electronic medical record
GP: General practitioner
GPMP: GP Management plan
HF: Heart failure
HFrEF: Reduced ejection fraction heart failure (EF < 50%)
HFpEF: Preserved ejection fraction heart failure (EF ≥ 50%)
HFuEF: Unquantified ejection fraction heart failure
JVP: Jugular venous pressure
LRTI: Lower respiratory tract infection
NT-proBNP: N terminal pro-brain natriuretic peptide
PHN: Primary Health Network
PND: Paroxysmal nocturnal dyspnoea
PIP: Practice Incentives Program
QI: Quality Improvement
URTI: Upper respiratory tract infection
