# Supplementary material for: The epidemiology of heart failure in the general Australian community - study of heart failure in the Australian primary carE setting (SHAPE): methods
Source: BMC Public Health. 2020 May 11;20:648. doi: 10.1186/s12889-020-08781-8 (PMC7216401; doi:10.1186/s12889-020-08781-8)
Supplement: Supplementary file 1 — Additional file 1. Appendix - Free text search terms. [file 12889_2020_8781_MOESM1_ESM.docx]

## Appendix - Free text search terms

The words or phrases searched for in the free text (clinical notes in the medical records). If any of the following free-text search terms was found and not surrounded with one of the ‘exclusion’ words or phrases, then the variable called HF_symp = 1 (which means that heart failure is classified as present in the free text notes). Note that in the following, the underscore character (_) represents a space. Where it is present before and after a set of characters, it ensures that the characters are not part of a larger word, but stand as a separate word

- DYSPNOEA
- DYSPNEA
- _PND_
- _SOB_
- _SOBOE
- SHORT and BREATH (note: the ‘and’ means both words need to appear close to each other)
- ORTHOPNOEA
- ORTHOPNEA
- PAROXYS and NOCTURNAL and (DYSPNOEA or DYSPNEA)
- FAILURE and (HEART or CARDIAC)
- CCF_
- CHF_
- HEART and CONGEST
- FAILURE and VENTRICULAR
- SYSTOLIC and DYSFUNCTION
- DIASTOLIC and DYSFUNCTION
- _HFREF_
- _HFPEF_
- OEDEMA and PULMONARY
- ODEMA and PULMONARY
- CARDIOMYOPATHY and HYPERTROP
- CARDIOMYOPATHY and (ISCHAEMIC or ISCHEMIC)
- CARDIOMYOPATHY and DILAT

For these last 3 terms, if the first one is found (hypertrop cardiomyopathy), then the other 2 were set to absent.

To exclude instances where HF terms were not present, in all cases the following phrases or words were sought in the 30 characters before or after the word (if it was found).:

- _NO_
- _NOT_
- _NIL_
- FAMILY HISTORY
- _NIL HISTORY
- _FAMILY HX
- _DENIES_
- _DENIAL_

The following terms were searched for in a similar manner:

- REDUCED EF
- EF REDUCE
- REDUCED EJECT
- EJECTION FRACTION REDUCE

If any of these last 4 terms is found, then the variable EF_Scan is set to 1 (positive mention of EF)

Other words which were sought in a similar manner are as follows:

- _JVP_ and (ELEVAT or HIGH or RAISE)
- _VENOUS PRESSUR
- JUGULAR and (HEPATO or REFLUX)
- _NECK_ and (DISTEND and VEIN)
- HEART SOUND and (THIRD or 3RD or _ADDED_)
- RHYTHM and (GALLOP or GALOP or GALLUP)
- _APEX BEAT_ and LATERAL and DISPLACE
- _APEX BEAT_ and VOLUME and LOAD
- ENLARGED and HEART
- COUGH and NOCTURNAL
- BENDOPNOEA
- BENDOPNEA
- LEAN and FORWARD and (DYSPNOEA or DYSPNEA or _SOB_)
- LEAN and FORWARD and (SHORT and BREATH)
- LEAN and FORWARD and BREATHLES
- WEIGHT and GAIN and 2 and (MORE or >)
- _OEDEMA_ and (PERIPHERAL or ANKLE or SACRUM or PITTING or _LEG_ or LOWER LIMB or DEPENDENT
- EDEMA_ and (PERIPHERAL or ANKLE or SACRUM or PITTING or _LEG_ or LOWER LIMB or DEPENDENT
- SWELLING and (ANKLE or _LEG_ or LOWER LIMB)
- CREPITATIONS_ or RALES or PULMONARY OEDEMA or PULMONARY EDEMA or FLUID OVERLOAD or LEFT VENTRICULAR DECOMPENS or CARDIAC DECOMPENS
